# An Analysis of Key Actor Networks for Scale-Up Strategies for Childhood Obesity Prevention and the Care of Children with Obesity in Brazil

**DOI:** 10.1016/j.cdnut.2023.101961

**Published:** 2023-06-03

**Authors:** Juliana Gonçalves Machado, Gabriela Buccini, Elisabetta Recine

**Affiliations:** 1 Human Nutrition Graduate Program, School of Health Science, University of Brasília (UnB), Federal District, Brazil; 2 Department of Social and Behavioral Health, School of Public Health, University of Nevada Las Vegas, Las Vegas, NV, United States

**Keywords:** childhood obesity, opinion leader, nutrition policy, implementation science, Net-Map

## Abstract

**Background:**

Effective scale-up of multisectoral strategies aimed to prevent and treat childhood obesity has been a challenge in Brazil, the largest country in Latin America. Implementation Science methods, such as Net-Map, can identify key actors and opinion leaders (OLs) to advance the implementation and promote sustainability.

**Objectives:**

This study aimed to analyze power relations between key actors and OLs who influence the scale-up of Brazilian strategies for childhood obesity at the federal and state/municipal (local) levels.

**Methods:**

A mixed method study, applying the Net-Map method, collected data through virtual workshops with federal and local level stakeholders. The Net-Map included key actors mapping, power mapping, and identification of OLs. Four domains of power were analyzed: command, funding, technical assistance, and dissemination. Network cohesion and centrality measures were calculated. A qualitative analysis was conducted to qualify power relations according to ∗ gears for a successful scale-up (i.e., coordination, goals, and monitoring; advocacy; political will; legislation and policy; funding and resources; training; program delivery; communication; and research and technical cooperation).

**Results:**

A total of 121 federal key actors and 63 local key actors were identified across networks, of which 62 and 28 were identified as OLs, respectively. Whereas the command domain of power had the highest number of key actors, the funding domain had the least. The health sector executive branch emerged as an OL across all domains of power.

**Conclusions:**

Barriers that threatened successful scale-up include the lack of coordination between domains of power, missing leadership within key actors, and lack of mechanisms to manage conflict of interest. Governance strategies to enhance multisectoral coordination and communication are needed to effectively scale-up and sustain childhood obesity strategies in Brazil.

## Introduction

Childhood obesity affects 39 million children under 5 y old worldwide [1]. According to the WHO, children aged <5 y with weight-for-height more than 3 SDs and children and adolescents from 5 to less than 19 y of age with BMI-for-age more than 2 SDs have obesity. It is a chronic and complex condition influenced by social ecological factors that integrates biological, social, personal, and environmental factors [2], reduces the quality of life for children with obesity in the long term [3,4], and increases healthcare costs [5]. Evidence-based recommendations to implement childhood obesity multisectoral strategies exist [4]; however, the global progress to reduce childhood obesity remains a challenge [6]. The World Obesity Federation report indicated that several countries, including Brazil, have low chances of meeting the WHO target of *no increase in obesity prevalence by 2025* [7].

The prevalence of childhood obesity in Brazil has increased [8] and reached 13.2% of the children from 5 to 9 y old monitored in the Primary Health Care service in 2019 [9]. Although Brazil is a global example of success in scale-up of child nutrition programs, such as the promotion of breastfeeding [10,11] and school feeding programs [12,13], the implementation of childhood obesity strategies has only started in the last 5 y [11].

In recent years, 2 national programs focused on childhood obesity have been created, and they are expected to help the effective reduction of childhood obesity. The first, the *Programa Crescer Saudável* (Healthy Growing Program), targets children enrolled in Early Childhood Education (daycare and preschools) and Elementary School [14]. The second, the *Estratégia Nacional para a Prevenção e Atenção à Obesidade Infantil* (PROTEJA, National Strategy to Prevent Childhood Obesity and to Promote Healthier Cities), is implemented in the context of Sistema Único de Saúde (Brazilian Universal Unified Health System). PROTEJA has a complex scope, which involves a set of multisectoral actions that encompass food and nutritional education, mass educational campaigns, training of professionals from different sectors (social assistance, education, health), and improving the food environment. PROTEJA is implemented at the municipal level but assumes a multisectoral commitment from federal, state, and municipal levels to prioritize the childhood obesity agenda [15].

Scale-up strategies allow the reach and effectiveness of actions to be maximized, leading to a sustained impact on outcomes for larger segments of the target population [16,17]. Scaling-up these and other multisectoral childhood obesity prevention strategies in Brazil must take into account the diverse social ecological context and implementation capacity of the 5570 Brazilian municipalities. Therefore, for a successful scale-up, municipalities must develop their own implementation plans following federal, state, and local policy decision-making roadmaps to fight childhood obesity inequities.

Scale-up of nutrition-related strategies has gained momentum globally due to the importance of operationalizing strategies in different contexts in an equitable and integrated manner [17,18]. Scaling up childhood obesity multisector programs is not an easy task, and several implementation barriers have been reported [4,19]. Therefore, recent evidence recommends the use of Implementation Science (IS) methods to support the effective implementation of strategies to prevent childhood obesity and care for children with obesity [20]. IS provides the perspective of applying scientific methods to promote greater effectiveness in the implementation of strategies in health services [21]⁠. One of the IS methods is the Net-Map, a participatory network mapping method that combines *1*) Stakeholder Mapping, *2*) Network Social Analysis, and *3*) Power Mapping. The Net-Map is used to understand the existing power relations in the decision-making process and produces a theoretical basis to support changes in the public policy scenario [22,23]. The Net-Map has been employed to strengthen the scale-up of infant feeding and nutrition strategies in Nepal [24], Mexico [25], and Bangladesh, Ethiopia, and Vietnam [26], based on the identification of key actors involved in the breastfeeding network in these countries⁠. Identifying and qualifying the role of the key actors involved and the established power relations can also help direct efforts to implement strategies related to childhood obesity.

Curbing childhood obesity implies the implementation of multisectoral strategies articulated with different key actors [4]. However, in Brazil, little is known about these actors, their intentions, and the nuances of their actions. Studies show that the role played by opinion leaders (OLs) who advocate for an agenda is crucial to a successful implementation of health promotion strategies at the community level [27]. OLs are individuals with the ability to influence the opinions, attitudes, beliefs, motivations, and behaviors of others. Their presence on an agenda can be favorable for it to be considered a priority [27]. This includes creating opportunities for action and allowing greater articulation and coordination of the policy processes [17]. Therefore, training new OLs is crucial to advance the infant feeding agenda as well as the formulation and execution of infant nutrition policies [28]. Knowing who the OLs are and to which organizations they belong could optimize implementing strategies focused on the childhood obesity agenda.

We aimed to analyze the power relations between key actors and OLs who influence the scale-up of Brazilian strategies for the prevention of childhood obesity and the care of children with obesity at the federal and state/municipal (local) levels. The use of IS, based on the Net-Map method, is justified to help reduce the gap in studies about scale-up strategies and analyze the connection networks of the childhood obesity agenda and the OLs involved in Brazil.

## Methods

### Study design

This exploratory mixed-method study with a convergent triangulation used qualitative data analysis to confirm the findings of the quantitative analysis [29]. This project was approved by the Research Ethics Committee of the School of Health Sciences of the University of Brasília (CAAE no. 47861721.6.0000.0030). All participants provided verbal consent following a description of the study’s purpose and design.

### Key actor analysis

A key actor analysis was conducted using Net-Map, a method developed by the International Food Policy Research Institute to identify and visualize a network with several key actors and to increase the understanding of the interactions between them to achieve a goal [22,30]⁠.

The present key actor analysis followed 3 activities: *1*) Key Actor Mapping (identifies key actors who influence childhood obesity agenda), *2*) Power Mapping (indicates key actors’ power within analyzed domains of power), and *3*) OL Identification (identifies OLs who embrace childhood obesity agenda) into 4 domains of power: command, funding, technical assistance, and dissemination; defined in Table 1.

**TABLE 1:** Key terms and domains of power

Key terms	Definitions
Stakeholders	Individuals or organizations who have an interest in an issue to be addressed, whether they are actively or potentially involved in affecting the outcomes of the policy in question. They may include important staff in ministries from sectors relevant to nutrition and other development partners (UN agencies, civil society organizations, donors, private sector groups, or community organizations) [30]⁠
Key actor	Individual or organization that makes decisions about implementing policies [26]⁠
Opinion leaders (OLs)	Individuals with the ability to influence the opinions, attitudes, beliefs, motivations, and behaviors of others [27]⁠
Governance system	Multisectoral cooperation, vertical coordination, and civil society engagement or mobilization, along with other factors, needed within the country to translate policy recommendations into action [17]⁠
Decision-making	The process involved in translating policy recommendations into action [53]⁠
Power	Level of influence that an individual or organization has to effect change [25]⁠
Domain of power	Field of endeavor in which an individual or organization exercises power, such as commanding and technical assistance [26]⁠
Domain of power of command	Key actor with connections to provide or receive commands about a schedule or task (example: a manager who can tell an employee to do a certain task) [25]⁠
Domain of power of funding	Key actor with connections to provide or receive financial resources or incentives (example: one key actor finances another’s projects) [26]⁠
Domain of power of technical assistance	Key actor with connections to provide or receive support in developing the technical assistance of people directly involved in the implementation of actions (example: one key actor offers training opportunities to another) [26]⁠
Domain of power of dissemination	Key actor with information-disseminating connections (example: 2 key actors that disseminate information about what one or both developed or supported) [26]⁠

### Identification of study participants

A convenience sampling of the stakeholders involved in the childhood obesity agenda was carried out [31]. Stakeholders were identified at federal, state, and municipal management levels of the Executive and Legislative branches, in addition to academia, civil society, and international organizations.

**At the federal level**, a list of potential participants was identified based on materials published by the Brazilian Ministry of Health (MS, Ministério da Saúde). Three specialists on childhood obesity—one from the Food and Nutrition Coordination of the Brazilian MS, one from Brazilian academia, and one from a civil society organization—were invited to rank the stakeholders from the list considering their perspective of the power each stakeholder has on the agenda. Stakeholder selection considered the power level of the organization, the group organization (e.g., government agency, academic organization), and the role (e.g., promotion, research, and evaluation). A total of 16 eligible study participants were invited through email to enroll in the study.

**At the local level**, a list of potential states and municipalities was identified based on their engagement in a national strategy to enhance childhood obesity through multisectoral actions. A group of specialists on the implementation of childhood obesity strategies from the Brazilian MS identified 6 eligible states according to *1*) being proactive primary health care management who works in the food and nutrition agenda and *2*) being representative of North/Northeast, Central-West, and South/Southeast regions. The highest rate of childhood obesity in primary healthcare in 2019 was used as an additional criterion to narrow down the participants to 3 states. The food and nutrition manager of these states helped to identify eligible participants at the municipal level based on the following criteria:

1. A representative of the legislative branch at the municipal or state level working on the agenda of childhood obesity;
2. A representative of a municipality with challenges implementing multisectoral childhood obesity strategies; and
3. A representative of a municipality with fewer challenges implementing multisectoral childhood obesity strategies.

From this, representatives from 6 municipalities were selected, and 2 congressmen were selected. Selected participants were invited via email. Appendix A presents the list of the participants enrolled in the study.

### Data collection

The research team, composed of the coauthors and an assistant, was trained prior to the Net-Map workshops through 2 online training sessions using materials translated and adapted from Buccini et al. [25]. Key terms were defined during the training (Table 1) as well as the role of each member in the workshop: coordination of workshops, registration of information, support for chat registration, and field notes.

**FIGURE 1:**
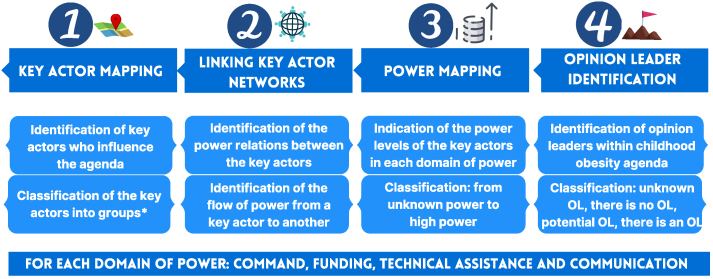
Steps taken for the Net-Map method. Source: Adaptation from Buccini et al. [26]⁠. ∗Key actors’ groups: executive branch, legislative branch, judicial branch, academic, media, civil society, private sector, international organization, and S system.

Data collection occurred from August 2021 to January 2022. The Net-Map virtual workshop happened in 2 meetings and lasted 3 h for each level (federal and local), following the steps illustrated in Figure 1. In step 1, participants were asked to identify key actors involved in the childhood obesity agenda. Then, the identified key actors were classified into groups. In step 2, participants were asked to link key actor networks using arrows to illustrate power relations from each other. In step 3, for the power mapping, participants were asked to rank each key actor’s power level to determine the extent to which each organization has the power to influence policy and programming decision-making. In step 4, OLs were identified by the participants. The Net-Map steps were repeated for 4 domains of power to build 4 Net-Map *per* workshop. The meetings were recorded, and workshop notes were taken for later analysis.

Due to time constraints, during the virtual workshops, the power mapping and the OL identification (steps 3 and 4) were adapted to collect data asynchronously or through an individual interview. Detailed instructions on how to complete the information were sent to the participants. A total of 11 (84.6%) participants at the federal level and 5 (50%) at the local level responded to these 2 Net-Map steps. In addition, the federal workshop did not have time to complete the power of dissemination domain; therefore, this specific domain was excluded from the federal analysis.

### Data sources and data management

Four data sources were managed in this study. The data from the Net-Map were organized into 2 data sheets for federal and local levels: *1*) *A spreadsheet of the key actors organized by group:* lists the characteristics of the identified key actors, that is, the name of the key actor and group that it participates for each level (e.g., executive branch, organized civil society). From this worksheet, the power level of each key actor was quantified (e.g., high power, medium power) for each domain of the power network, and the OLs were identified (e.g., there is an OL, there is no OL). *2*) *A spreadsheet of the links*: indicates the source and target key actor of the links established for each domain of power. These spreadsheets were used to generate *3*) the *Net-Map*. In addition, *4*) *verbatim transcripts of the workshops and field notes* from the team were systematized to be used as data sources.

### Data analysis

**FIGURE 2:**
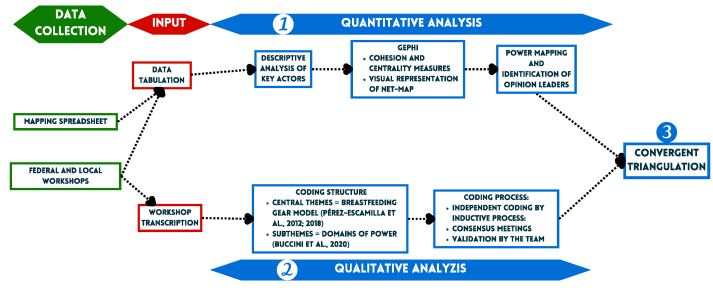
Illustration of the analysis approach applied to the present study. Source: The authors.

Data analysis was conducted in three steps: *1*) quantitative analysis, *2*) qualitative analysis, and *3*) data triangulation (Figure 2).

#### Quantitative analysis

The spreadsheets were imported into the Gephi 0.9.6 program to generate a directed Net-Map using the Yifan Hu algorithm. The domains of power were used as the unit of analysis, resulting in a Net-Map for each power domain at the federal and local levels.

A descriptive analysis of the key actors from the federal and local levels was carried out for each Net-Map. First, the size of the domain of the power network was described, including: *1*) the number of key actors, *2*) the frequency of groups of key actors (e.g., executive branch, civil society), and *3*) the number of links established (e.g., State Health Department sends a link to the Minister of Health).

Power relations between the key actors for each Net-Map were analyzed according to the cohesion and centrality measurements, as defined in Table 2 [26,32,33]. Cohesion measures (i.e., density and distance) reflect the interconnectivity of key actors within the domain of power. Regarding the density, although it can be difficult to estimate the cohesiveness or fragmentation of a network, the range from 0.30 to 0.50 was considered appropriate [34]. The distance was analyzed in a relative way, comparing the networks of the domains of power. Centrality measures (i.e., mean degrees, in-degree centrality, out-degree centrality, and betweenness centrality) reflected the most prominent key actors in the domains of power [25]. To streamline the analysis, a ranking of 5 key actors with the highest values of centrality measures for each domain of power was presented for the federal and local levels.

**TABLE 2:** Measures of cohesion and centrality for social network analysis

Term	Definition
Distance	Average number of links between nodes. Where distances are great, it may take a long time for information to diffuse across a population; moreover, key actors who are closer to more people may be able to exert more power than those who are more distant
Density	The proportion of links actually present out of all possible links. Density is a ratio that can range from 0 to 1. The closer to 1 the density, the more interconnected the network
Mean degrees	Average number of links that pass through the nodes, identifying the key actors in the network
In-degree centrality	Measures the number of links directed at a key actor, representing the input received from a particular network
Out-degree centrality	Measures the number of links from a key actor directed to other key actors in the network
Betweenness centrality	Measures the number of times a key actor connects subgroups within a network. Represents the capacity of an organization to control the flow of information in the network between any pair of the other organizations in the policy network. It is assumed that the *middle actors* have more *interpersonal influence* over others in the network

**Source:** [26,33]**⁠**

Power levels of each key actor were determined by calculating the mode across participants’ responses ranging from 0 (unknown power), 1 (no power), 2 (little power), 3 (medium power), to 4 (high power). Similarly, the OLs were determined by ranking each key actor from 0 (unknown leadership), 1 (there is no OL), 2 (there could be an OL), or 3 (there is an OL). Key actors with mode = 3 were considered OLs. Detailed information about the OLs identified can be found in Appendix B.

In the Net-Map, each key actor was indicated by a node, and the connection to one another was represented by arrows. Key actors who were not connected were excluded from the analysis. The nodes were color-coded according to key actor groups: yellow (executive branch), light blue (legislative branch), purple (judiciary branch), light green (academia), pink (media), dark blue (civil society), red (private sector), dark green (international organizations), and gray (system S). The S system covers organizations of corporate entities focused on professional training, social assistance, consulting, research, and technical assistance, such as Social Service of Commerce (SESC – Serviço Social do Comércio) and National Industrial Apprenticeship Service (SENAI – Serviço Nacional de Aprendizagem Industrial). The diameter of the node proportionally represents the measure of betweenness centrality.

#### Qualitative analysis

For the thematic analysis, the workshops were transcribed verbatim*,* and the field notes were reviewed [35]⁠. The analysis was developed using the following steps:

Definition of the coding structure: to guide the analysis, the coding structure was defined a priori with themes and subthemes. The themes were defined based on an adaptation of the interconnected gears for successful scale-up of child nutrition programs (i.e., coordination, goals, and monitoring; advocacy; political will; legislation and policies; funding and resources; training and program delivery; promotion; and research and evaluation) [10,36]. Pérez-Escamilla et al. [10,36] describe that interconnected gears work as a machine in which advocacy is necessary to generate the political will to influence legislation and policies focused on the agenda, which are essential for the government to allocate budget and resources for program training and for program delivery, in addition to promoting the agenda to the population. Research and program evaluation are also important to maintain its effectiveness and quality. By managing the entire operation, there is a central gear responsible for goals coordination and feedback of the process [37]. In this study, Training and Program Delivery were split into 2 gears. Operational definitions for each gear, themes, and subthemes were adapted to the context of the childhood obesity agenda in Brazil.⁠

Coding: Workshop transcriptions were independently read line by line by 2 team members (JGM and GB). Themes were highlighted, classified within the predefined coding structure, and coded into sub-subthemes through an inductive process. The codebook was established through a consensus process [35] in which 2 members discussed and aligned the codes in weekly meetings. The final version of the codebook was reviewed through an iterative process by the senior research members (GB and ER) to clarify and agree upon the meaning and definition of the themes and subthemes. The frequency of each theme and subtheme, as well as the sub-subthemes, were summarized. Findings from the thematic analysis are indicated by the acronyms of the gears/themes (Table 3) throughout the results section.

**TABLE 3:** Definition of the themes and subthemes of the thematic analysis based on the Breastfeeding Gear Model [10,36] and the domains of power [26]

Theme	Definition
Advocacy (A)	Indicates some effort to translate evidence-based recommendations into advocacy actions to advance the childhood obesity agenda. Uses social mobilization strategies to engage people, decision-makers, and resources to generate pressure and influence political will
Political will (PW)	Indicates the presence or absence of a government’s express institutional and budgetary commitment to implement a policy. Used whenever the context is at the configured schedule level
Legislation and policy (LP)	Related to the establishment and enactment of national laws, rules, regulations, and policies on childhood obesity that demonstrate commitment to expand, promote, and support programs and initiatives on the subject
Funding and resources (FR)	Related to the discussion of budget and funding strategies that demonstrate commitment to expanding childhood obesity programs
Training (T)	Regarding the training offered to professionals responsible for implementing the strategies of the agenda on attitudes, knowledge, and skills in counseling and managing childhood obesity
Program delivery (PD)	Pertaining to activities planned and carried out at all levels of health care, including programs in health services and community-based programs
Communication (C)	Indicates the use of a variety of methods (including social media, national and local events, campaigns, community activities, and soft skills) to communicate messages about childhood obesity to the general population
Research and technical cooperation (R)	Regarding actions that involve the production of scientific evidence to support technical, normative, or popular documents on childhood obesity programs, in a way that favors the sharing of information and allows appropriate decision-making for implementation, at each level, in a timely manner
Coordination, goals, and monitoring (CM)	Related to the synchronization and integration of activities, responsibilities, and command and control structures to ensure that public resources are used in the most efficient manner to adequately fulfill the policy function of preventing childhood obesity and caring for children with obesity

**Subtheme**	**Definition**

Domain of power of command	Related to the level of influence that an individual or organization has to command an agenda or task
Domain of power of funding	Related to the level of influence that an individual or organization has to provide financial resources
Domain of technical assistance	Related to the level of influence that an individual or organization has to support the development of technical assistance of people directly involved in the implementation of actions
Domain of power of dissemination	Related to the level of influence that an individual or organization has to disseminate information about childhood obesity

#### Convergent triangulation

Convergent triangulation of quantitative and qualitative data was employed [29]. Despite greater emphasis on quantitative analysis, as it directly involves mapping power relations, qualitative data helped deepen and qualify the findings about the relationships as well as identify barriers and facilitators of the context in which the powers were identified.

## Results

### Identification of key actors, opinion leaders, and themes

**FIGURE 3:**
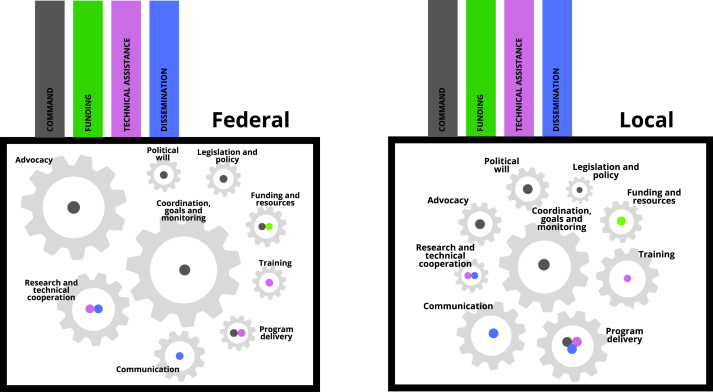
Maps of key actors involved in Brazilian childhood obesity strategies sized by betweenness centrality, color coded by groups of key actors and stratified by domains of power and level of action: command links (A and B), funding links (C and D), technical assistance links (E and F), and dissemination links (G). Legends: Colors of stakeholder groups: yellow (executive branch), light blue (legislative branch), purple (judiciary branch), light green (academia), pink (media), dark blue (civil society), red (private sector), dark green (international organizations), and gray (system S). Stakeholder size (node size) is proportional to the betweenness measures. Domains of power: Tech Assist (technical assistance). Key actors: ACADEMIA, key actors academia condensed; CAISANE, State Multisectoral Council of Food and Nutrition Security; CGAN, Food and Nutrition Coordination of the Brazilian Ministry of Health; HCPRO, healthcare professionals; SES, State Department of Health; SMS, Municipal Department of Health.

A total of 189 key actors were identified at the federal level and 69 at the local level. Of these, 121 federal (64.0%) and 63 local (91.3%) key actors were linked to another key actor across the domain of power. Regarding the size of the networks, 448 links were identified at the federal level and 416 at the local level, which were characterized by the domains of power (Figure 3).

**FIGURE 4:**
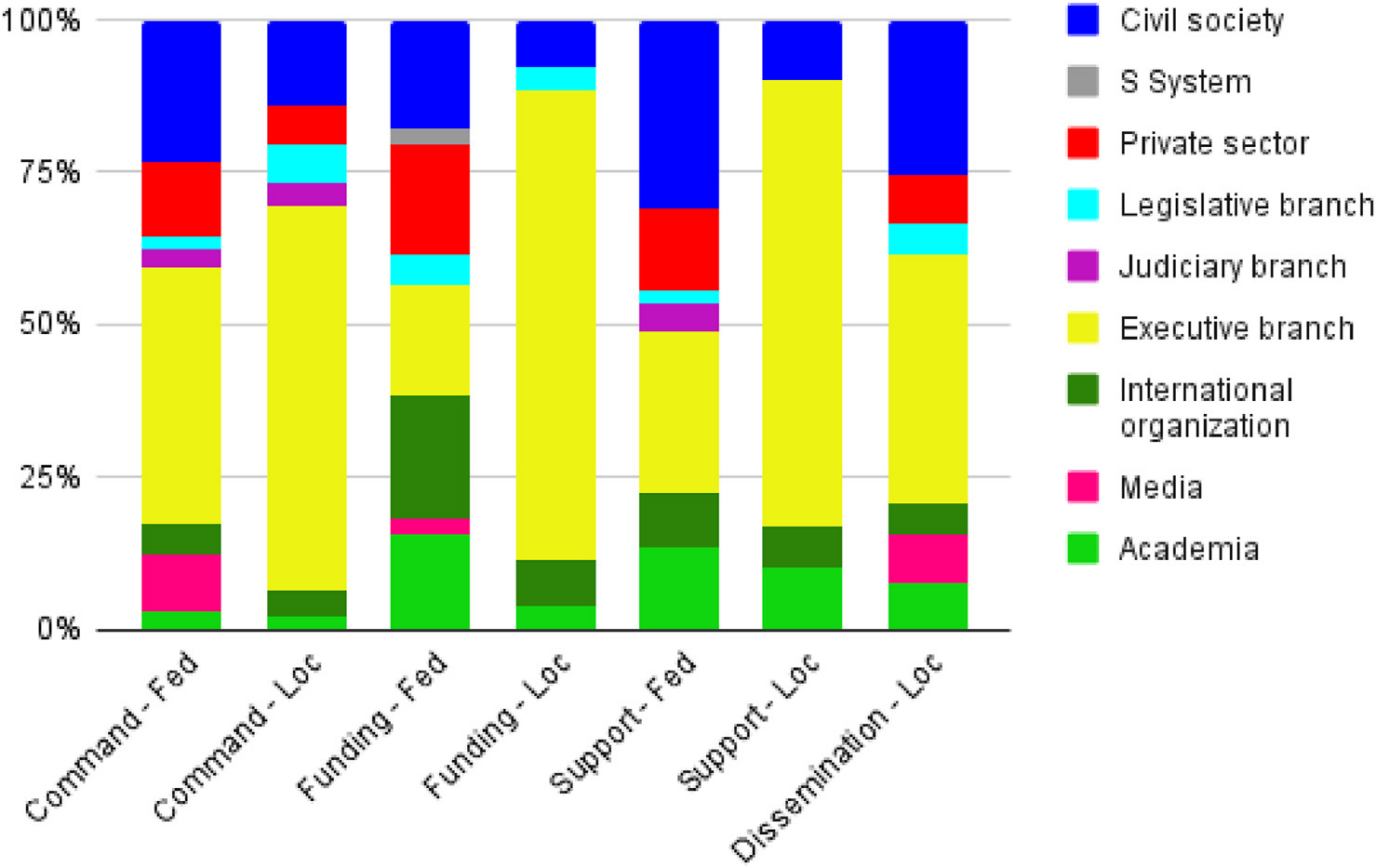
Prevalence of key actor groups through federal and local Net-Map distributed according to domains of power. Source: The authors. Legend: Loc, Local; Fed, Federal; Support, technical assistance.

The distribution of the key actor groups across the 4 domains of power is presented in Figure 4. The results of the power mapping are presented by group of key actors in Appendix C. The Ministry of the Economy and some key actors in the legislative branch were classified as having high power for at least one domain of power; however, they did not have links in any domain of power.

A total of 95 key actors were identified as OLs at the federal level and 29 at the local level. Of those, 62 OLs at the federal level and 28 at the local level were linked to another key actor(s) in at least one domain of power; therefore, they were included in the Net-Map analysis. The majority of OLs were from the executive branch (18 federal and 17 local OLs) followed by the civil society (16 federal and 6 local OLs).

**FIGURE 5:**
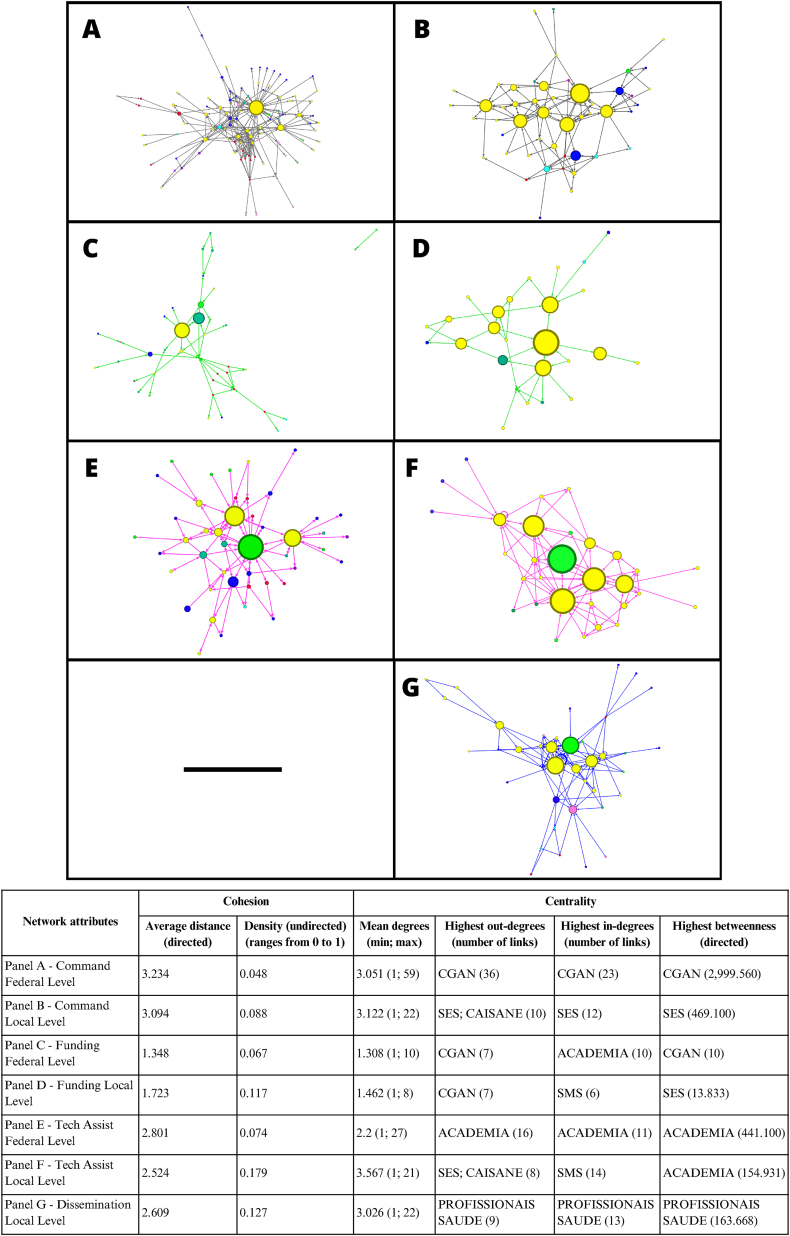
The proposed model combines analysis of the domains of power [26] and the Breastfeeding Gear Model [10]. Source: The authors. Color legends: gray (domain of power of command), green (domain of power of funding), violet (domain of power of technical assistance), and blue (domain of power of dissemination). Gear size (node size) is proportional to the number of sub-subthemes identified in the thematic analysis.

The qualitative analysis indicates that at least one domain of power (subtheme) is acting in each gear (theme). In our analysis, the gears were found to be the core of the scale-up machine and the domains of power are the driving force to make each gear spin. In Figure 5, the colors represent the domains of power responsible for spinning the gears, and the size of the gears is proportional to the number of citations per theme identified in the thematic analysis. The main gears functioning, at the federal level, are advocacy, coordination, goals, and monitoring as well as research and technical cooperation. The main gears functioning, at the local levels, are training, program delivery, and communication, as well as coordination, goal, and monitoring.

### Integration of the results

In the next section, the convergent qualitative and quantitative results from the Net-Map analysis are presented for each of the 3 domains of power (Figure 4).

### Domain of power of command

The command domain of power was composed of a network of 98 key actors and 299 links at the federal level, and a network of 49 key actors and 153 links at the local level.

Regarding cohesion measures, network density (d) was low, and the distance (ad) was relatively high for both federal (d = 0.048; ad = 3.234) and local levels (d = 0.088; ad = 3.094), with the executive branch standing out in the rankings (TABLE 4, TABLE 5). Paradoxically, this was the analyzed domain of power with the highest number of OLs at both the federal (*n* = 53) and local levels (*n* = 23), mostly from the executive branch and civil society groups. In the qualitative analysis, bureaucratic relations (Coordination, goals, and monitoring gear – CM), decision-makers with conflict of interest (Political will gear – PW), and lack of leadership of strategic key actors for the agenda, such as ministers, governors, and mayors (PW) were identified as barriers to effective command and to scale up the strategies.

**TABLE 4:** Integration of results for the domain of power of command at the federal level

Ranking of the key actors with the highest centrality actions				Role of the key actor according to the BFGM of Pérez-Escamilla et al. [10]⁠	Mapping of powers of command domain and identification of OLs
Key actor	B	OD	ID		
CGAN	2999.560	36	23	CM: Leadership of CGAN CM: Bureaucratic relations	OL (+) with high power
FNDE	1080.702	14	12	PW: Agenda Pullers CM: Horizontal integration CM: Bureaucratic Relations	OL (+) with high power
CGPAE	533.688	–	–	CM: Horizontal integration CM: Bureaucratic Relations	OL (+) with high power
DICOL	518.534	–	15	CM: Horizontal integration CM: Regulation CM: Bureaucratic Relations	OL (+/−) with high power
MINMS	448.520	–	–	PW: Important for the agenda, but without leadership CM: Relationship with the Minister	OL (+) with high power
GGFIS	–	13	13	CM: Horizontal integration CM: Regulation CM: Bureaucratic Relations	OL (+) with medium power
GGALI	–	12	13	CM: Horizontal integration CM: Regulation CM: Bureaucratic Relations	OL (?) with high power
ALIANCA	–	12	–	A: Advocacy strategies of civil society A: Leadership to define the agenda	OL (+) with medium power

**Key actors:** ALIANCA, Alliance for Adequate and Healthy Food; CGAN, Food and Nutritional Coordination of the Ministry of Health; CGPAE, General Coordination of the National School Nutrition Program of the Ministry of Education; DICOL, Collegiate Board of Directors of National Health Regulatory Agency, Anvisa; FNDE, National Education Development Fund; GGALI, General Food Management, National Health Regulatory Agency, Anvisa; GGFIS, General Management of Sanitary Inspection and Supervision of Anvisa; MINMS, Minister of Health.

**Centrality measures**: B, Betweenness; ID, In-degree; OD, Out-degree.

**BFGM (Breastfeeding Gear model):** A, Advocacy; C, Communication; CM, Coordination, goals, and monitoring; FR, Funding and resources; LP, Legislation and policy; PD, Program delivery; PW, Political will; R, Research and technical cooperation; T, Training.

**Identification of Opinion Leaders:** OL (+) [there is an opinion leader], OL (+/−) [there could be an opinion leader], OL (−) [there is no opinion leader], OL (?) [unknown].

**TABLE 5:** Integration of results for the domain of power of command at the local level

Ranking of the key actors with the highest centrality measures				Role of the key actor according to the BFGM of Pérez-Escamilla et al. [10]⁠	Mapping of powers of the command domain and identification of OLs
Key actor	B	OD	ID		
SES	469.100	10	12	CM: Leadership of the SES	OL (+) with high power
SEDUEST	341.583	8	11	CM: Horizontal integration	OL (+) with medium power
SMS	305.267	–	8	CM: Vertical Integration	OL (+) with high power
CAISANE	279.967	10	8	CM: Spaces for multisectoral articulation	OL (+/−) with unknown power
UBS	278.717	9	8	PD: APS as network coordinator	OL (+/−) with little power
COAN	–	9	–	CM: Local governance CM: Horizontal integration CM: Vertical Integration	OL (+) with medium power

**Key players:** CAISANE, State Multisectoral Council of Food and Nutrition Security; COAN, State Food and Nutritional Coordination; SEDUEST, State Education Department; SES, State Department of Health; SMS, Municipal Health Department; UBS, Basic Healthcare Centers.

**Centrality measures**: B, Betweenness; ID, In-degree; OD, Out-degree.

**BFGM (Breastfeeding Gear model):** A, Advocacy; C, Communication; CM, Coordination, goals, and monitoring; FR, Funding and resources; LP, Legislation and policy; PD, Program delivery; PW, Political will; R, Research and technical cooperation; T, Training.

**Identification of Opinion Leaders:** OL (+) [there is an opinion leader], OL (+/−) [there could be an opinion leader], OL (−) [there is no opinion leader], OL (?) [unknown].

The Food and Nutrition Coordination of the Brazilian Ministry of Health and the State Department of Health are the OLs with the highest power of command at their respective levels (TABLE 4, TABLE 5). Both were classified as the coordinators, leading the vertical and horizontal integration of strategies related to childhood obesity (CM). However, the actions of these OLs depend on the political will of key actors in higher hierarchical positions, such as ministers, and governors, who were not identified as OLs for the childhood obesity agenda (PW). Medium and low-power key actors were described as articulators of childhood obesity at the local level, although their performance was classified as low by the participants (PW). Horizontal integration is a challenge due to bureaucratic barriers (CM). In qualitative analysis, the creation of management tools such as management reports has been used to improve the planning, assessment, and monitoring of childhood obesity strategies (CM).

The lack of key actors from the judiciary and legislative branches was clear in the thematic analysis (Legislation and policy gear - LP) and in their absence on TABLE 4, TABLE 5. On the other hand, the private sector was perceived to have high negative power on the domain of power of command at the federal and local levels (Advocacy gear - A), although it was not in the command ranking. The civil society key actors tended to have less power to influence the childhood obesity agenda, and their connections had only few OLs with high power, especially at the local level; however, qualitative analysis classified civil society as a critical group for inducing and pressuring decision-makers about the childhood obesity agenda (A).

### Domain of power of funding

The domain of power of funding consisted of 39 key actors and 51 links at the federal level, and 26 key actors and 38 links at the local level. Regarding cohesion measures, the network had low density, although the distance was the lowest among the domains of power analyzed at federal (d = 0.067; ad = 1.348) and local levels (d = 0.117; ad = 1.723). The ranking of key actors at the federal level consisted of a variety of key actor groups (Table 6); in contrast, the local level ranking consisted majority of key actors from the executive branch (Table 7).

**TABLE 6:** Integration of results for the domain of power of funding at the federal level

Ranking of the key actors with the highest centrality measures				Role of the key actor according to the BFGM of Pérez-Escamilla et al. [10]⁠	Mapping of powers of power of funding domain and identification of OLs
Key actor	B	OD	ID		
CGAN	10	7	–	FR: Government funding of research FR: Government funding for key actors of civil society	OL (+) with high power
PAHO	7	3	–	FR: Research funding mediated by international organizations FR: Research funding of projects by international organizations	OL (+) with medium power
FIOTEC	3	–	–	FR: Government funding of research (operator)	OL (?) with unknown power
IDEC	2	–	–	FR: Funding by civil society	OL (+) with little power
CNPQ	1	–	3	FR: Government funding of research (operator)	OL (?) with high power
DICOL	–	6	–	FR: Government funding of research	OL (+/−) with medium power
FNDE	–	5	–	FR: Government funding of research (potential)	OL (+) with high power
ABIA, ABIR	–	3	–	FR: Research Funding by the Private Sector FR: Funding by lobbyists	OL (+) with high power
IPA	–	3	–	FR: Funding by lobbyists FR: Funding from legislative sources	OL (?) with unknown power
ACADEMIA	–	–	10	FR: Government funding of research (receiver) FR: Research Funding by the Private Sector (receiver)	OL (+) with unknown power
LOBBY	–	–	6	FR: Funding by lobbyists	OL (?) with unknown power
FIOCRUZ	–	–	3	FR: Government funding of research (receiver)	OL (+) with medium power
NGO	–	–	3	FR: Government funding for key actors of civil society	OL (+) with unknown power

**Key actors:** ABIA, Brazilian Food Industry Association; ABIR, Brazilian Association of Soft Drink and Non-Alcoholic Beverage Industries; ACADEMIA, key actors in academia condensed; CGAN, Food and Nutritional Coordination of the Ministry of Health; CNPQ, National Council for Scientific and Technological Development; DICOL, Anvisa Collegiate Board of Directors; FIOCRUZ, Oswaldo Cruz Foundation; FIOTEC, Foundation for resource management; FNDE, National Education Development Fund; IDEC, Consumer Defense Institute; IPA, Instituto Pensar Agropecuária; LOBBY, lobbyists; NESTLE, Nestle; NGO, organizations of general civil society; PAHO, Pan American Health Organization.

**Centrality measures**: B, Betweenness; ID, In-degree; OD, Out-degree.

**BFGM (Breastfeeding Gear model):** A, Advocacy; C, Communication; CM, Coordination, goals, and monitoring; FR, Funding and resources; LP, Legislation and policy; PD, Program delivery; PW, Political will; R, Research and technical cooperation; T, Training.

**Identification of Opinion Leaders:** OL (+) [there is an opinion leader], OL (+/−) [there could be an opinion leader], OL (−) [there is no opinion leader], OL (?) [unknown].

**TABLE 7:** Integration of results for the domain of power of funding at the local level

Ranking of the key actors with the highest centrality measures				Role of the key actor according to the BFGM of Pérez-Escamilla et al. [10]⁠	Mapping of the power of funding domain and identification of OLs
Key actor	B	OD	ID		
SES	13.833	2	4	FR: Implementation of resources for program delivery	OL (+) with medium power
SMS	8.167	–	6	FR: Implementation of resources for program delivery	OL (+) with high power
CGAN	8.000	7	–	FR: Government funding of research FR: Funding of programs	OL (+) with high power
COAN	6.000	–	–	FR: Implementation of resources for program delivery	OL (+) with medium power
SASSISTEST	5.667	3	2	FR: Implementation of resources for program delivery	OL (+) with unknown power
MCID	–	4	–	FR: Funding of programs	OL (+/−) with medium power
CAISANE	–	4	–	FR: Funding of programs	OL (+/−) with no power
UNICEF	–	2	–	FR: Research funding mediated by international organizations	OL (+) with high power
GOV	–	2	–	FR: Implementation of resources for program delivery PW: Obstacle to the agenda	OL (-) with high power
MS	–	2	–	FR: Funding of programs	OL (+) with high power
CCM	–	2	–	FR: Congregational amendments to fund the agenda	OL (+/−) with unknown power
ACADEMIA	–	–	5	FR: Government funding of research (receiver)	OL (+/−) with no power
SEDUEST	–	–	3	FR: Implementation of resources for program delivery	OL (+) with unknown power
SEDUMUN	–	–	2	FR: Implementation of resources for program delivery	OL (+) with medium power
SASSISTMUN	–	–	2	FR: Implementation of resources for program delivery	OL (+/−) with no power
UBS	–	–	2	FR: Implementation of resources for program delivery (receptor)	OL (+/−) with no power
AF	–	–	2	FR: Implementation of resources for program delivery (receptor)	OL (+/−) with unknown power

**Key actors:** ACADEMIA, key actors Brazilian academia condensed; AF, Family Farmers; CAISANE, State Multisectoral Council of Food and Nutrition Security; CCM, City Councilmen; CGAN, General Food and Nutritional Coordination of the Ministry of Health; COAN, Food and Nutritional Coordination; GOV, Governor; MCID, Ministry of Citizenship; MS, Ministry of Health; SASSISTEST, State Department of Social Assistance; SASSISTMUN, Municipal Department of Social Assistance; SEDUEST, State Department of Education; SEDUMUN, Municipal Department of Education; SES, State Department of Health; SMS, Municipal Department of Health; UBS, Basic Healthcare Centers; UNICEF, United Nations Children’s Fund.

**Centrality measures**: B, Betweenness; ID, In-degree; OD, Out-degree.

**BFGM (Breastfeeding Gear model):** A, Advocacy; C, Communication; CM, Coordination, goals, and monitoring; FR, Funding and resources; LP, Legislation and policy; PD; Program delivery; PW, Political will; R, Research and technical cooperation; T, Training.

**Identification of Opinion Leaders:** OL (+) [there is an opinion leader], OL (+/−) [there could be an opinion leader], OL (−) [there is no opinion leader], OL (?) [unknown].

High power OLs from the private sector fund research and influence the agenda negatively at federal level (Funding and resources gear - FR). Qualitatively, the conflict of interest in the funding for research and lack of multisectoral funding for implementation of childhood obesity agenda were reported as implementation barriers (FR, CM). According to federal level participants, the source of funding will shape the content of the study product produced by ACADEMIA (key actors in academia condensed) (FR). High power OLs from the executive branch and civil society were able to fund research free of conflict of interest (FR). The Pan American Health Organization (Organização Pan-americana de Saúde) is a medium power key actor with high betweenness centrality, which qualitatively is translated by the key role in mediating research and implementation funding from the Brazilian MS to ACADEMIA and civil society groups (FR).

At the local level, the discussion centered on funding for implementing childhood obesity strategies (FR). Implementation funding is provided by key actors within the Brazilian MS and the Brazilian Ministry of Citizenship (Table 7). The Municipal Department of Health is a high-power key actor for receiving and executing funding (FR, CM). However, identified barriers to utilize the implementation funds were bureaucracy and lack of political will and leadership of high power key actors such as governor, mayor, and municipal health secretary (FR, PW).

### Domain of power of technical assistance

The domain of power of technical assistance consisted of a total of 45 key actors and 98 links at the federal level and 30 key actors and 107 links at the local level.

We identified a low density (d = 0.074) in the federal network, with a distance of 2.801 between key actors (Table 8). At the local level, this network had the highest density (d = 0.179) among the domains of power analyzed; however, the distance was 2.524 (Table 9), which qualitatively may indicate that the implementation of strategies could take longer to be executed.

**TABLE 8:** Integration of results about the power of domain of technical assistance at the federal level

Ranking of the key actors with the highest centrality measures				Role of the key actor according to the BFGM of Pérez-Escamilla et al. [10]⁠	Mapping the power of the development of technical assistance domain and identification of OLs
Key actor	B	OD	ID		
ACADEMIA	441.100	16	11	T: Technical assistance institutions PD: Academia to support delivery R: Production of technical materials and documents to qualify implementation R: Production with conflict of interest R: Leaders of research to monitor and evaluate production of materials	OL (+) with high power
CGAN	336.067	9	9	R: Production of technical materials and documents to qualify implementation	OL (+) with high power
FNDE	282.950	11	6	R: Production of technical materials and documents to qualify implementation	OL (+) with high power
ACT	145.400	–	–	R: Leaders of research to monitor and evaluate production of materials A: Advocacy strategies of civil society	OL (+) with high power
GGALI	101.450	6	5	R: Production of technical materials and documents to qualify implementation	OL (?) with high power
UNICEF	–	6	-	R: Production of technical materials and documents to qualify implementation	OL (+) with high power
HCPRO	–	–	5	T: Training with conflict of interest (target audience) PD: In school programs PD: Breastfeeding programs R: Production of technical materials and documents to qualify implementation (target audience)	OL (+/−) with high power

**Key actors:** ACADEMIA, key actors in academia condensed; ACT, ACT Health Promotion; CGAN, Food and Nutritional Coordination of the Ministry of Health; FNDE, National Fund for Education Development; GGALI, General Food Management, National Health Regulatory Agency, Anvisa; HCPRO, healthcare professionals from the health network; UNICEF, United Nations Children’s Fund.

**Centrality measures**: B, Betweenness; ID, In-degree; OD, Out-degree.

**BFGM (Breastfeeding Gear model):** A, Advocacy; C, Communication; CM, Coordination, goals, and monitoring; FR, Funding and resources; LP, Legislation and policy; PD, Program delivery; PW, Political will; R, Research and technical cooperation; T, Training.

**Identification of Opinion Leader:** OL (+) [there is an opinion leader], OL (+/−) [there could be an opinion leader], OL (−) [there is no opinion leader], OL (?) [unknown].

**TABLE 9:** Integration of results about the domain of power of technical assistance domain at the local level

Ranking of the 5 key actors with the highest centrality measures				Role of the key actor according to the BFGM of Pérez-Escamilla et al. [10]⁠	Mapping the power of the development of technical assistance domain and identification of OLs
Key actor	B	OD	ID		
ACADEMIA	154,931	6	–	T: Technical assistance institutions T: Teaching and Service Integration PD: Academia to support delivery P: Production of technical materials and documents to qualify implementation	OL (+/−) with high power
SMS	135,272	6	14	T: Technical assistance institutions	OL (+) with medium power
SES	124,767	8	13	T: Technical assistance institutions T: Teaching and Service Integration PD: International Organizations that support delivery (target audience)	OL (+) with high power
UBS	112,168	–	10	T: Integration of Teaching and Service T: Technical assistance institutions (target audience) PD: in-school programs	OL (+/−) with little power
CAISANE	97,083	8	–	T: Technical assistance institutions	OL (+/−) with little power
CGAN	–	7	–	T: Technical assistance institutions P: Production of technical materials and documents to qualify implementation	OL (+) with high power
COAN	–	6	7	T: Technical assistance institutions	OL (+) with high power
PSEMUN	–	6	–	T: Technical assistance institutions	OL (+) with high power
HCPRO	–	–	10	T: Integration of Teaching and Service T: Technical assistance institutions (target audience) T: Matrix-based strategies by PHC professionals for the agenda	OL (+) with high power

**Key actors:** ACADEMIA, key actors in academia condensed; CAISANE, State Multisectoral Council of Food and Nutrition Security; CGAN, Food and Nutritional Coordination of the Ministry of Health; COAN, Food and Nutritional Coordination; HCPRO, Healthcare professionals; PSEMUN, Municipal Managing Committee of School Healthcare; SES, State Department of Health; SMS, Municipal Health Department; UBS, Basic Healthcare Centers.

**Centrality measures**: B, Betweenness; ID, In-degree; OD, Out-degree.

**BFGM (Breastfeeding Gear model):** A, Advocacy; C, Communication; CM, Coordination, goals, and monitoring; FR, Funding and resources; LP, Legislation and policy; PD, Program delivery; PW, Political will; R, Research and technical cooperation; T, Training.

**Identification of Opinion Leader:** OL (+) [there is an opinion leader], OL (+/−) [there could be an opinion leader], OL (−) [there is no opinion leader], OL (?) [unknown].

ACADEMIA is a high power key actor at both federal and local levels; however, it is an OL only at the federal level (TABLE 8, TABLE 9). At the federal level, ACADEMIA is tasked to qualify advocacy in partnership with key actors from the executive branch (Fundo Nacional de Desenvolvimento da Educação [National Education Development Fund], Gerência-Geral de Alimentos da Anvisa [General Food Management]) (A, Research and technical cooperation gear - R) and enhance the monitoring system of program delivery (Table 8); at the local level, it provides technical assistance and opportunities for continuing education to equip human resources for successful implementation and delivery (Table 9).

Healthcare professionals (HCPROs) are high power key actors at both federal and local levels. They receive technical assistance links from other key actor groups, such as the executive branch, academia, and international organizations. They were also responsible for training other professionals (Training gear - T) and for executing the strategies in the communities (Program delivery - PD). In parallel, the Basic Healthcare Centers (where the healthcare professionals [HCPROs] are housed) are low power key actors with links consisting of institutionalized processes for continuing education and academic internship.

### Domain of power of dissemination

The domain of power of dissemination consisted of a network of 39 key actors and 118 links at the local level; this domain of power was not discussed at the federal level. Regarding cohesion measures, at the local level, the network was found low density (d = 0.127) and a distance of 2.609 between key actors. About 64.1% (*n* = 25) were considered high power key actors, with the majority of them being from the executive branch (*n* = 11, 44%).

The HCPRO was identified as a high-power OL with the highest centrality measures within the dissemination network (Table 10). Qualitatively, the HCPRO plays a critical role in the dissemination during the delivery of programs, as well as exchanging information among HCPROs from other healthcare teams (Communication gear - C).

**TABLE 10:** Integration of results for the power of domain of dissemination at the local level

Ranking of the 5 key actors with the highest centrality measures				Role of the key actor according to the BFGM of Pérez-Escamilla et al. [10]⁠	Mapping of power to disseminate information domain and identification of OLs
Key actor	B	OD	ID		
HCPRO	163,668	9	13	PD: Performance of PHC professionals in program delivery C: Communication Strategy of the Executive Branch	OL (+) with high power
ACADEMIA	158,588	7	–	C: Dissemination strategies on the agenda WITH conflict of interest R: Production of information	OL (+/−) with high power
SES	108,169	–	11	C: Communication Strategy of the Executive Branch C: Dissemination strategies on the agenda WITHOUT conflict of interest	OL (+) with high power
UBS	99,183	7	11	PD: Performance of PHC professionals in program delivery	OL (+/−) with little power
SMS	71,356	–	–	C: Communication Strategy of the Executive Branch	OL (+) with medium power
COAN	–	7	–	C: Communication Strategy of the Executive Branch C: Dissemination strategies on the agenda WITHOUT conflict of interest C: Dissemination strategies on the agenda WITH conflict of interest	OL (+) with high power
NASF	–	7	–	PD: Performance of PHC professionals in program delivery	OL (+/−) with high power
NGO	–	7	–	C: Dissemination strategies on the agenda WITHOUT conflict of interest R: Production of information	OL (?) with little power
COMM	–	7	–	C: Dissemination strategies on the agenda WITH conflict of interest	OL (+) with high power
PATRONS	–	–	11	PD: Performance of PHC professionals in program delivery (target audience)	OL (+/−) with no power
SOCM	–	–	8	C: Media and influence on families and/or healthcare professionals C: Industry marketing and influence on families and/or healthcare professionals	OL (+/−) with high power

**Key actors:** ACADEMIA, key actors in academia condensed; COAN, State Coordination of Food and Nutrition; COMM, Commercial Representatives; HCPRO, healthcare professionals; NASF, Family Health Support Centers; NGO, Civil society NGOs; PATRONS, Patrons; SES, State Department of Health; SOCM, Social Media; UBS, Basic Healthcare Centers.

**Centrality measures**: B, Betweenness; ID, In-degree; OD, Out-degree.

**BFGM (Breastfeeding Gear model)**: A, Advocacy; C, Communication; CM, Coordination, goals, and monitoring; FR, Funding and resources; LP, Legislation and policy; PD, Program delivery; PW, Political will; R, Research and technical cooperation; T, Training.

**Identification of Opinion Leaders:** OL (+) [there is an opinion leader], OL (+/−) [there could be an opinion leader], OL (−) [there is no opinion leader], OL (?) [unknown].

High power OLs from the executive branch carried out communication strategies on childhood obesity for professionals (C). ACADEMIA is an OL with high power to collect, produce, and disseminate information (R). Potential conflicts of interests were identified in the dissemination links between ACADEMIA and the private sector (C). Likewise, conflicts of interest were identified in dissemination links between key actors from social media and from the private sector (C).

## Discussion

To our knowledge, this is the first study to use IS methods to investigate health-related governance systems for childhood obesity in Brazil. The use of the domains of power and the 9 gears for a successful scaling up as frameworks revealed the key actors and the OLs across the networks according to domains of power in addition to how their role in the networks enabled the scaling up of the childhood obesity agenda in Brazil. A parallel can be drawn between scaling up a strategy and a machine [10]. The machine can best perform its tasks when all gears are present, and the pistons work in harmony. In turn, the NetMap's domains of power are the pistons that move the gears according to the force exerted by the power relations between the key actors involved in the networks. Power relations can facilitate or hinder the operation of gears, depending on who has greater power to move them. Finally, OLs can be compared to machine lubricating oils, as their presence catalyzes machine movement. We found that both federal and local *machines* have the potential to work properly because all gears are present when there are key actors working in each gear and OLs to facilitate the functioning. However, machines were found to work inefficiently due to the low strength of the relationship between the key actors. Factors that might be behind this malfunctioning may be conflicting forces, power imbalance among key actors, and lack of leadership among key actors in strategic positions, especially at the local level. Moreover, our study highlights opportunities to improve the Brazilian environment for the prevention of childhood obesity and the care of children with obesity.

Our analysis indicated that the federal and local levels play different roles in the scaling up of childhood obesity agenda. The federal level sets the agenda—which is the prioritization of a health issue to become a policy [38]—for the scale-up through which high power key actors from executive branch influence and are influenced by key actors from academia, civil society, and private sector groups. On the other hand, the local level is where the agenda is implemented depending on the political will locally, which is mostly induced by the federal funding and the OLs in the executive branch. This behavior is related to the gears’ performance on the scaling up according to the levels of governance. While, at the federal level, advocacy and research and technical cooperation were critical; at the local levels, the focus was on training, program delivery, and communication. Coordination, goal, and monitoring gear stood out in the discussion at both levels, which reinforces the involvement of OLs in roles related to this gear. Having a strong coordination gear drives multisectoral articulation and communication between different levels, which was a key to the success of national breastfeeding programs [39] and may be key for the childhood obesity agenda as well.

Agenda-setting depends on the intention of key actors involved [38]; therefore, some concerns regarding conflict of interest were raised about the private sector’s high power in funding training, program delivery, research, scientific events, and social media. Conflict of interest is defined as a set of conditions in which professional judgment concerning a primary interest tends to be unduly influenced by a secondary interest [40]. Corroborating our findings, prior studies have identified conflicts of interest in the relationship between OLs from the legislative and executive branches and the private sector as a barrier to implementing the childhood obesity agenda in Brazil [41,42]. Mechanisms through legislation and policy to identify, manage, and prevent conflicts of interest may be an important step to strengthen the cohesion in the networks for scaling up sustainable strategies for the childhood obesity agenda [43].

Our study identified a relatively small and low cohesive funding network with the executive branch at the federal level being the main funding source for the childhood obesity agenda. Analyses from other countries identified similar results of size and cohesion; however, their composition varied. For example, while in Mexico the results were similar to our study [26], in Bangladesh, international and civil society organizations were protagonists in funding programs related to infant and young child feeding, with little active involvement from government key actors [32]. Our results reinforce that challenges faced by developing countries in managing public budgets and financing programs related to nutrition are similar [44]. As an alternative, mechanisms to enhance budget control and decision-making, such as transferring resources to the local level and participatory budgeting, have been shown to promote cohesiveness and speed decision-making for child development services [45].

A strong body of OLs within the health sector was identified coordinating the agenda at the federal level. Strong coordination has been critical to articulate sectors, determine goals, and manage obstacles during the scaling-up [17,18,46,47]. Corroborating our findings, prior studies demonstrate the presence of technical healthcare areas in the leadership of strategies related to child food and nutrition [26,32,48]. On the other hand, the lack of powerful OLs in the local level coordination can delay multisectoral cohesion for implementation [49]. Thus, leadership training targeting multisectoral key actors may promote the cohesion of the local governance necessary for scaling up and sustainability of the childhood obesity agenda.

In our study, HCPROs were identified as OLs due to their role in program delivery and communication. A well-trained OL with a close connection to a community can organize an action plan [48] and adapt interventions to the context [49], which increases the chances of effectiveness and sustainability⁠. Therefore, training HCPROs can optimize program delivery in the childhood obesity agenda, given that they work directly with users of public services [50,51]. Key actors from academia and civil society groups can help provide this training [52].

We acknowledge that our study has some limitations that should be considered when interpreting the results presented in this work. First, there have been changes in federal and state governments, as well as federal and state deputies due to the elections in 2022, which may lead to some changes in the classification of power level and OLs of the key actors identified in the near future. Second, a methodological limitation is that the present study represents a specific perspective of the stakeholders interviewed in the workshops, according to their experiences and perspectives. On the other hand, we selected participants from different organizations, which allowed us to capture a comprehensive perception of the networks due to the fact that participants were familiar with different aspects of the childhood obesity agenda. The systematic preparation of data collection and the mixed methods with integration of results from quantitative and qualitative data sources can be considered as an innovation and strengthen the findings that would not be possible with a traditional approach.

In summary, our findings identified OLs from different groups and sectors across the domains of power at the federal and local levels, which should have a positive impact on the social ecological perspective of the childhood obesity agenda. Nevertheless, the leadership of the healthcare sector is critical to generate the political will necessary to move the scaling-up machine. Increasing multisectoral leadership, enhancing coordination, and determining goals are key to achieve effective scale-up of the childhood obesity agenda in Brazil.

Our mixed-method study identified a complex governance system to scale up the agenda for childhood obesity prevention and care of children with obesity. We identified strengths and opportunities to enhance cohesion across domains of power networks and ultimately reduce implementation barriers. Planning and implementing strategies for the childhood obesity agenda might involve a detailed action plan, with specific roles for each key actor across the network, providing an appropriate environment for articulation, commitment, and accountability. These strategies along with strong coordination and monitoring are critical to keep the scaling-up machine working properly.

Nevertheless, scaling up the childhood obesity agenda needs to consider social ecological factors involved in each context; thus, based on our findings, we recommend:

• Improving network cohesion and horizontal and vertical integration of strategies to build more efficient governance of childhood obesity prevention strategies and caring for children with obesity;
• Strengthening civil society and academia by generating the political will of key actors from the legislative and executive branches;
• Using a tool to prevent and manage conflicts of interest in nutrition programs to reduce the power of the private sector in the political will on the agenda, such as the tool proposed by the Pan American Health Organization [43]⁠;
• Prioritizing key actors from the other sectors who need to be informed and, therefore, involved and committed to the agenda;
• Prioritizing specific funds for multisectoral strategies to prevent childhood obesity and to care for children with obesity, including investing in a regulatory agenda to improve the food environment and mass media campaigns;
• Investing in OL training in management at the local level, especially in sectors other than healthcare;
• OL training among HCPROs for better implementation of strategies by facilitating the delivery of programs with greater efficiency and effectiveness.

## Funding

This study was supported by the Ministry of Health of Brazil through a Pan American Health Organization project. The supporting source had no involvement or restrictions regarding publication.

## Author disclosures

ER reports financial support provided by Ministry of Health of Brazil through a Pan American Health Organization project. JGM and GB report no conflicts of interest.

## Data availability

Data described in the manuscript, code book, and analytic code will be made available upon request through email to the corresponding author.

## Appendix A Supplementary data

Multimedia component1 Multimedia component1
