# Supplementary material for: An Analysis of Key Actor Networks for Scale-Up Strategies for Childhood Obesity Prevention and the Care of Children with Obesity in Brazil
Source: Curr Dev Nutr. 2023 Jun 3;7(7):101961. doi: 10.1016/j.cdnut.2023.101961 (PMC10310469; doi:10.1016/j.cdnut.2023.101961)
Supplement: Multimedia component1 [file mmc1.docx]

**SUPPLEMENTARY DATA**

**Title of the article**

# An analysis of key actor networks for scale-up strategies for childhood obesity prevention and the care of children with obesity in Brazil

# **Corresponding Author’s name, mailing address, telephone number, and email address**

Juliana Gonçalves Machado

Rua Visconde de Figueiredo, 99, Tijuca, Rio de Janeiro - RJ, Brazil. Apartment 403

+55 81 995769690

[machado.julig2@gmail.com](mailto:machado.julig@gmail.com)

# APPENDIX A – Workshop participants

| **Federal Level** | | |
| --- | --- | --- |
| **Stakeholder group** | **Number of participants** | **Federal representatives** |
| Executive branch | 6 | - Food and Nutritional Coordination of the Ministry of Health (Coordenação-Geral de Alimentação e Nutrição – CGAN/MS) - Coordination for the Promotion of Physical Activity and Intersectoral Actions, Ministry of Health (Coordenação de Promoção de Atividade Física e Ações Intersetoriais – CGPROFI/MS) - Coordination of Child Health and Breastfeeding, Ministry of Health (Coordenação de Saúde da Criança e Aleitamento Materno – COCAM/MS) - National Education Development Fund, Ministry of Education (Fundo Nacional de Desenvolvimento da Educação – FNDE/MEC) - General Food Management, National Health Regulatory Agency, Anvisa (Gerência Geral de Alimentos, Agência Nacional de Vigilância Sanitária, Anvisa – GGALI) |
| Academia | 2 | - Department of Social Nutrition at the State University of Rio de Janeiro (Departamento de Nutrição Social da Universidade do Estado do Rio de Janeiro – UERJ) - Department of Nutrition at the Federal University of Minas Gerais (Departamento de Nutrição da Universidade Federal de Minas Gerais – UFMG) |
| Civil society | 3 | - Pastoral da Criança - Brazilian Institute of Consumer Defense (Instituto Brasileiro de Defesa do Consumidor – IDEC) - ACT Health Promotion (ACT Promoção da Saúde) |
| International organization | 2 | - Pan American Health Organization (Organização Pan-Americana da Saúde – PAHO) - United Nations Children's Fund Brazil (Fundo das Nações Unidas para a Infância Brasil – Unicef Brasil) |
| **Local Level** | | |
| **State** | **Number of participants** | **Local representatives** |
| Mato Grosso do Sul | 3 | - State technical reference for food and nutrition - Nutritionist at PHC* in the municipality of Fátima do Sul - PHC coordinator in the municipality of Iguatemi |
| Pernambuco | 3 | - State technical reference for food and nutrition - PHC nutritionist in the municipality of Orocó - PHC coordinator in the municipality of Macaparana |
| Rio Grande do Sul | 4 | - State technical reference for food and nutrition - State health regional coordinator - Nutritionist in the municipality of Ibirubá (works at different points in the healthcare network) - PHC coordinator for the municipality of Colorado |
| **Source: The authors**  *PHC = Primary Health Care | | |

# APPENDIX B - Opinion leaders distributed according to key actor groups

| **Groups of Key actors** | **Frequency of the identification of opinion leaders** | | | | | | | | | |
| --- | --- | --- | --- | --- | --- | --- | --- | --- | --- | --- |
|  | **Federal** | | | | | **Local** | | | | |
|  | **OL** | **Could be OL** | **No OL** | **Unknown** | **Total** | **OL** | **Could be OL** | **No OL** | **Unknown** | **Total** |
| Academia | 5 | 0 | 0 | 5 | 10 | 1 | 2 | 0 | 0 | 3 |
| Media | 7 | 0 | 0 | 2 | 9 | 0 | 2 | 0 | 1 | 3 |
| International Organization | 8 | 0 | 0 | 1 | 9 | 2 | 0 | 0 | 0 | 2 |
| Executive Power | 18 | 10 | 0 | 17 | 45 | 17 | 18 | 1 | 3 | 36 |
| Judicial power | 0 | 2 | 0 | 1 | 3 | 0 | 1 | 0 | 1 | 2 |
| Legislative power | 2 | 1 | 0 | 0 | 3 | 0 | 1 | 2 | 0 | 3 |
| Private Sector | 6 | 0 | 0 | 7 | 13 | 2 | 0 | 1 | 0 | 3 |
| S System | 0 | 1 | 0 | 0 | 1 | 0 | 0 | 0 | 0 | 0 |
| Civil Society Organization | 16 | 4 | 0 | 8 | 28 | 6 | 4 | 0 | 1 | 11 |
| Total | 62 | 0 | 18 | 41 | 121 | 28 | 25 | 4 | 6 | 63 |
| **Compiled by authors** | | | | | | | | | | |
| **Legends of Identification of Opinion Leaders: OL** (There is an OL); **Could be OL** (There could be an OL); **No OL** (There is no OL); **Unknown** (Unknown leadership) | | | | | | | | | | |

**APPENDIX C - Tables of federal and local level power mappings**

| Groups of Key actors | **Power level in the domain of power of command** | | | | | | | | | | | |
| --- | --- | --- | --- | --- | --- | --- | --- | --- | --- | --- | --- | --- |
|  | **Federal** | | | | | | **Local** | | | | | |
|  | **UN** | **NO** | **S** | **M** | **L** | **T** | **UN** | **NO** | **S** | **M** | **L** | **T** |
| Academia | 1 |  | 1 | 1 |  | 3 |  |  |  |  | 1 | 1 |
| Media | 3 |  | 4 | 1 | 1 | 9 |  |  |  |  |  | 0 |
| International Organization |  |  |  |  | 5 | 5 |  |  |  |  | 2 | 1 |
| Executive Power | 12 |  | 2 | 6 | 20 | 40 | 5 |  | 7 | 6 | 13 | 31 |
| Judicial power |  |  |  | 1 | 2 | 3 | 1 |  |  |  | 1 | 2 |
| Legislative power |  |  |  |  | 2 | 2 | 1 |  | 1 | 1 |  | 3 |
| Private Sector | 1 |  |  | 4 | 7 | 12 |  |  |  | 2 | 1 | 3 |
| S System |  |  |  |  |  |  |  |  |  |  |  |  |
| Civil Society Organization | 6 |  | 7 | 10 | 1 | 24 | 1 |  | 4 | 2 |  | 7 |
| Total | 23 |  | 14 | 23 | 38 | 98 | 8 | 0 | 12 | 11 | 18 | 49 |
| **Source: The authors** | | | | | | | | | | | | |

**Legends:**

**Power levels:** UN (Unknown or does not have power), NO (No power), S (Little power), M (Medium power), L (A lot of power), T (Total)

| Groups of Key actors | **Power level in the domain of power of funding** | | | | | | | | | | | |
| --- | --- | --- | --- | --- | --- | --- | --- | --- | --- | --- | --- | --- |
|  | **Federal** | | | | | | **Local** | | | | | |
|  | **UN** | **NO** | **S** | **M** | **L** | **T** | **UN** | **NO** | **S** | **M** | **L** | **T** |
| Academia | 3 |  | 1 | 1 | 1 | 6 |  | 1 |  |  |  | 1 |
| Media | 1 |  |  |  |  | 1 |  |  |  |  |  | 0 |
| International Organization | 3 |  |  | 3 | 2 | 8 |  |  |  |  | 2 | 2 |
| Executive Power |  | 1 | 1 | 1 | 4 | 7 | 5 | 3 | 1 | 4 | 7 | 20 |
| Judicial power |  |  |  |  |  |  |  |  |  |  |  | 0 |
| Legislative power |  |  |  | 1 | 1 | 2 | 1 |  |  |  |  | 1 |
| Private Sector | 3 |  |  |  | 4 | 7 |  |  |  |  |  |  |
| S System |  |  |  | 1 |  | 1 |  |  |  |  |  |  |
| Civil society organization | 2 |  | 4 | 1 |  | 7 | 1 | 1 |  |  |  | 2 |
| Total | 12 | 1 | 6 | 8 | 12 | 39 | 7 | 5 | 1 | 4 | 9 | 26 |
| **Source: The authors** | | | | | | | | | | | | |

**Legends:**

**Power levels:** UN (Unknown or does not have power), NO (No power), S (Little power), M (Medium power), L (A lot of power), T (Total)

| Groups of Key actors | **Power level in the domain of power of technical assistance** | | | | | | | | | | | |
| --- | --- | --- | --- | --- | --- | --- | --- | --- | --- | --- | --- | --- |
|  | **Federal** | | | | | | **Local** | | | | | |
|  | **UN** | **NO** | **S** | **M** | **L** | **T** | **UN** | **NO** | **S** | **M** | **L** | **T** |
| Academia | 2 |  |  |  | 4 | 6 |  |  |  |  | 3 | 3 |
| Media |  |  |  |  |  |  |  |  |  |  |  |  |
| International Organization | 1 |  |  |  | 3 | 4 |  |  |  |  | 2 | 2 |
| Executive Power | 1 |  |  | 1 | 8 | 10 | 4 | 1 | 3 | 4 | 10 | 22 |
| Judicial power | 2 |  |  |  |  | 2 |  |  |  |  |  |  |
| Legislative power |  |  | 1 |  |  | 1 |  |  |  |  |  |  |
| Private Sector | 1 |  |  | 5 |  | 6 |  |  |  |  |  |  |
| S System |  |  |  |  |  |  |  |  |  |  |  |  |
| Civil Society Organization | 8 | 1 | 1 | 1 | 4 | 15 |  |  | 2 |  | 1 | 3 |
| Total | 15 | 1 | 2 | 7 | 19 | 44 | 4 | 1 | 5 | 4 | 16 | 30 |
| **Source: The authors** | | | | | | | | | | | | |

**Legends:**

**Power levels:** UN (Unknown or does not have power), NO (No power), S (Little power), M (Medium power), L (A lot of power), T (Total)

| Groups of Key actors | **Power level in the domain of power of dissemination** | | | | | | | | | | | |
| --- | --- | --- | --- | --- | --- | --- | --- | --- | --- | --- | --- | --- |
|  | **Federal** | | | | | | **Local** | | | | | |
|  | **UN** | **NO** | **S** | **M** | **L** | **T** | **UN** | **NO** | **S** | **M** | **L** | **T** |
| Academia | NA | NA | NA | NA | NA | NA |  |  |  |  | 3 | 3 |
| Media | NA | NA | NA | NA | NA | NA |  |  |  |  | 3 | 3 |
| International Organization | NA | NA | NA | NA | NA | NA |  |  |  |  | 2 | 2 |
| Executive Power | NA | NA | NA | NA | NA | NA |  |  | 2 | 3 | 11 | 16 |
| Judicial power | NA | NA | NA | NA | NA | NA |  |  |  |  |  |  |
| Legislative power | NA | NA | NA | NA | NA | NA |  | 1 | 1 |  |  | 2 |
| Private Sector | NA | NA | NA | NA | NA | NA |  |  |  |  | 3 | 3 |
| S System | NA | NA | NA | NA | NA | NA |  |  |  |  |  |  |
| Civil society organization | NA | NA | NA | NA | NA | NA | 1 | 1 | 5 |  | 3 | 10 |
| Total | NA | NA | NA | NA | NA | NA | 1 | 2 | 8 | 3 | 25 | 39 |
| **Source: The authors** | | | | | | | | | | | | |

**Legends:**

**Power levels:** UN (Unknown or does not have power), NO (No power), S (Little power), M (Medium power), L (A lot of power), T (Total)
